# Design, Performance and Optimization for Multimodal Radar Operation

**DOI:** 10.3390/s120912673

**Published:** 2012-09-17

**Authors:** Surendra S. Bhat, Ram M. Narayanan, Muralidhar Rangaswamy

**Affiliations:** 1 Department of Electrical Engineering, The Pennsylvania State University, University Park, PA 16802, USA; E-Mail: ssb166@psu.edu; 2 Air Force Research Laboratory, Wright Patterson AFB, Dayton, OH 45433, USA; E-Mail: muralidhar.rangaswamy@wpafb.af.mil

**Keywords:** multimodal radar, adaptive radar, LFM, HRR profile, bandwidth optimization

## Abstract

This paper describes the underlying methodology behind an adaptive multimodal radar sensor that is capable of progressively optimizing its range resolution depending upon the target scattering features. It consists of a test-bed that enables the generation of linear frequency modulated waveforms of various bandwidths. This paper discusses a theoretical approach to optimizing the bandwidth used by the multimodal radar. It also discusses the various experimental results obtained from measurement. The resolution predicted from theory agrees quite well with that obtained from experiments for different target arrangements.

## 1. Need for Multimodal Radar

Increasingly complex target scenarios call for sophisticated techniques such as waveform and sensing diversity for resolving individual targets or target scattering centers to aid in target identification and recognition. Waveform design is therefore an essential ingredient of modern radar systems [1]. With the available electromagnetic (EM) spectrum becoming increasingly scarce, a crucial requirement for multimodal sensor operation is full adaptive waveform capability [2,3]. Different radar scenes have different resolution requirements for optimal target recognition, which translates into different bandwidths. Also, operating the radar at the highest bandwidth (and the best resolution) at all times has the disadvantage of requiring more processing and not leaving any bandwidth for other applications, such as communications or telemetry. To address these issues, a multimodal radar methodology has been proposed which can survey different target scenes with varying bandwidths [4,5]. The sharing of spectrum for radar and communications has been studied before [6,7].

Multifunction radio frequency (RF) systems have been studied for a long time [8–10]. A scalable multifunction RF system allows the RF functionality (radar, electronic warfare and communications) to be easily extended and the RF performance to be scaled to the requirements of different missions and platforms [8].

Multifunction radar systems have also been developed [11–14]. These are phased array systems that can perform a wide variety of functions such as tracking, surveillance, *etc.* [15]. More recently, the concept of cognitive radar was introduced [16]. Such a system consists of intelligent signal processing based on interactions between the radar and the environment, receiver-transmitter feedback to optimize transmit waveform parameters, and memory to facilitate target tracking using a Bayesian paradigm.

The multimodal radar has the ability to provide target indication with a large range extent and can progressively switch to a narrow range extent mode for extracting recognizable target features. Primary requirements for such a radar include detection and location of stationary targets in severe ground clutter as well as the classification and recognition of these targets.

A multimodal radar has been designed and developed by us to address the above needs. It consists of a test-bed that enables the generation of linear frequency modulation (LFM) waveforms with varying bandwidths. A narrow bandwidth waveform is used initially to obtain a low range resolution (LRR) profile of the target. High range resolution (HRR) processing is then progressively performed using higher bandwidth waveforms within selected range cells wherein targets are declared.

Radar resolution has been the focus of research for a very long time. Woodward applied the two-dimensional matched filter response to the analysis of radar resolution [17]. The ambiguity function was extended to include the co-ordinates of azimuth and elevation in [18]. Rihaczek concluded that the optimum radar signal for target resolution is the one that is matched to the environment [19]. In [20], the common definition used for measuring range resolution for equal strength targets was modified for targets of unequal strength. We develop a method to determine the optimum bandwidth for a target scene using convex optimization. We also look at the effect of targets of unequal radar cross section (RCS) over this bandwidth. The theoretical results are compared with the experimental data obtained from field measurements.

This paper is organized as follows: Section 2 provides a description of the multimodal radar and its operation. In Section 3, the bandwidth optimization procedure is presented with examples. The field measurement results for various scenarios are discussed in Section 4. In Section 5, we show results of extensive simulations to characterize the multimodal radar system. Conclusions are discussed in Section 6.

## 2. Multimodal Radar System Description

In this section, we discuss the system block diagram, various design parameters, and the flowchart of operation of the multimodal radar.

### 2.1. Block Diagram

**Figure 1.**
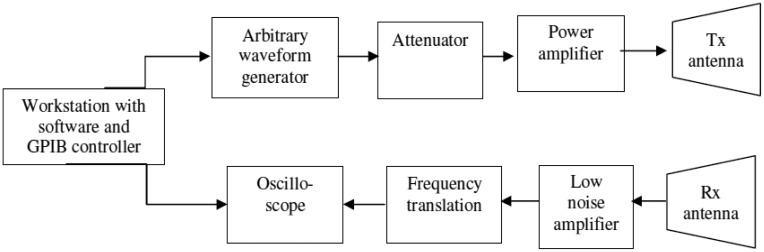
Notional block diagram of the multimodal radar.

Figure 1 shows the block diagram of the multimodal radar. The arbitrary waveform generator (AWG) enables generation of chirp waveforms of different bandwidths. The AWG operates at a maximum rate of 4 GSa/s, making it possible to generate waveforms of frequencies up to 2 GHz. The test-bed radar also includes amplifiers, transmitting and receiving antennas, and a high-speed oscilloscope for recording the received signal. The frequency spectrum of the transmitted signal lies within the 1,000–1,640 MHz band. Frequency translation is performed to downconvert the received signal from the 1,000–1,640 MHz band to the 300–940 MHz. The oscilloscope samples and records the return signal at 4 GSa/sec. The return is processed by software which decides whether further processing is required and what bandwidth waveform must be used for the next pass, if any. A workstation with a GPIB controller makes it possible for the software to control the AWG and the oscilloscope. Hence, the radar is capable of automatically making decisions about additional processing and required bandwidth. The radar system parameters are as shown in Table 1.

### 2.2. LRR and HRR Profiles

Initially, the AWG transmits a low bandwidth waveform (40 MHz) and sweeps the range extent searching for targets generating low range resolution profiles. Range resolution Δ*R* is given by *c*/2*B* where *c* is the speed of light and *B* is the bandwidth [21]. Thus, a bandwidth of 40 MHz corresponds to a range resolution of 3.75 m. The return is compared to an adaptive range-dependent detection threshold to obtain the LRR gates with a high probability of the existence of potential targets.

The multimodal radar now restricts its attention to those LRR gates where the threshold is exceeded. HRR imaging begins on these identified LRR gates with the 80 MHz bandwidth waveform (1.875-m resolution). Imaging stops if the desired range resolution is obtained on a particular LRR gate to identify existing targets, else it continues with the next higher bandwidth (160 MHz → 320 MHz → 640 MHz). Range resolution is progressively enhanced until a minimum separation (in dB) is met between the peaks and its neighboring cells. This minimum separation may be decided based upon the required resolution and the expendable bandwidth. Thus, the multimodal radar continues to look at potential targets with narrower range extents until the desired resolution is obtained to detect target presence. Table 2 summarizes the various bandwidths used by the multimodal radar and their corresponding range resolutions.

### 2.3. Flowchart for Operation in Staring Mode

**Figure 2.**
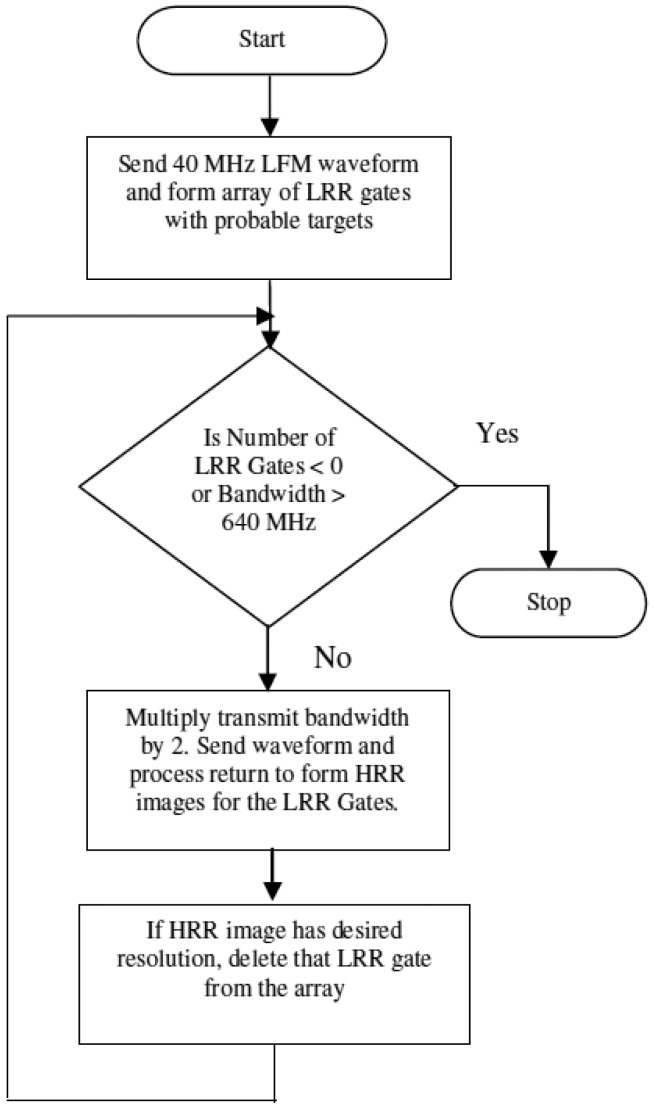
Flowchart for operation of multimodal radar in staring mode.

Figure 2 shows the flowchart for operation of the multimodal radar in staring mode, *i.e.*, with stationary antennas. It is assumed that the frequency band of 1–1.64 GHz is available for use. LRR gates are identified using a LFM waveform of bandwidth 40 MHz. Then the bandwidth is increased in powers of 2 until the desired resolution is obtained for each HRR profile. The simulation may require different number of passes for different LRR gates. The multimodal radar makes efficient use of the spectrum, utilizing lower bandwidths initially, and using the higher bandwidths only if required. This leaves the unused bandwidth available for use in other applications.

### 2.4. Flowchart for Operation in Scanning Mode

**Figure 3.**
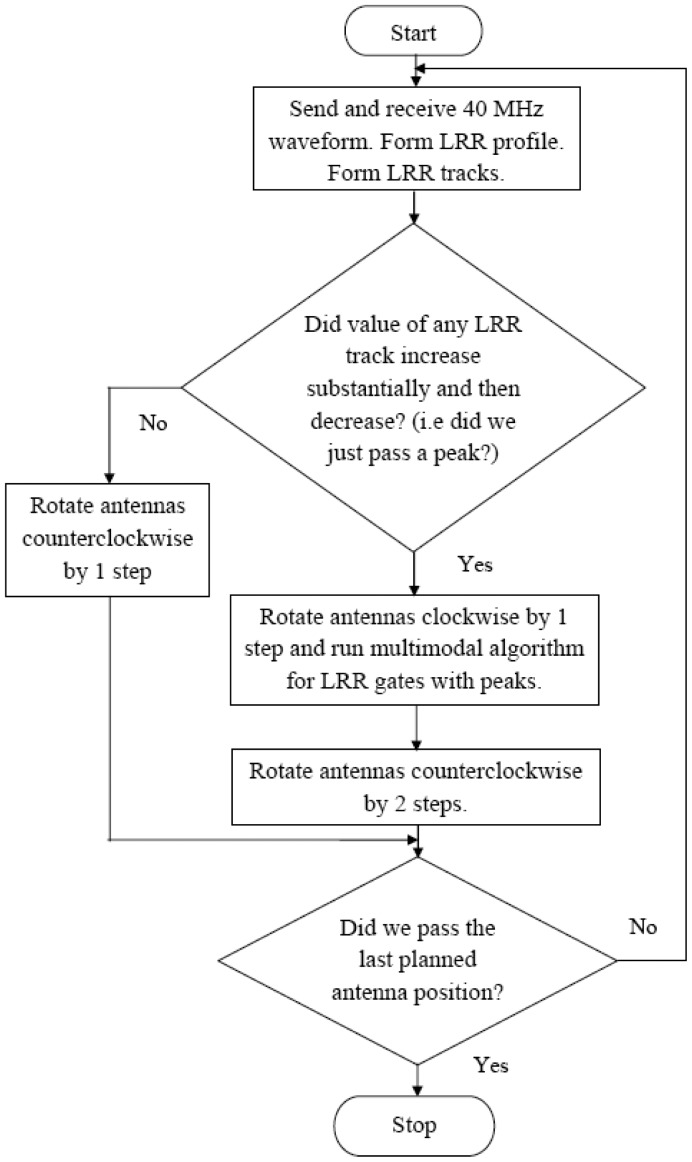
Flowchart for operation of multimodal radar in scanning mode.

Measurements were also taken for scenarios where multiple target scenes are laid out in azimuthal fashion. The radar starts scanning the field for targets (by rotating the antennas about a vertical axis) beginning with the low bandwidth waveform. The first LRR profile is taken at *φ* = 0 and subsequent ones are taken after rotating the antennas counterclockwise each time by a fixed angle, henceforth referred to as one step. The LRR profiles are stored to form LRR tracks for each LRR gate. When the radar detects that a peak has just been passed, the antennas are rotated clockwise (in the opposite direction) by one step to run the multimodal algorithm on the target scene. Figure 3 shows the flowchart for operation of the radar when scanning in this fashion. The antennas are mounted on a plate which can rotate in the azimuthal plane. The entire setup is automated so that the scanning process can operate without any user intervention.

## 3. Bandwidth Optimization

We consider the problem of determining the optimum bandwidth required for a target scene. The cost function for our optimization problem is the bandwidth which is same as the optimization variable. The separation between the peak and its neighboring cells can serve as the constraints. We would like to have 3-dB amplitude separations between the target peak and its neighboring cells. This should be adequate to provide a good representation of the range-related variability within the target scene. Using higher separation may increase the required bandwidth significantly.

### 3.1. Optimization problem

The optimization problem can be stated as below:

minimize *x*
subject to *f*_1_(*x*) ≥ 3 dB
  *f*_2_(*x*) ≥ 3 dB
where *x* is the bandwidth used for a target scene,
*f*_1_ is the separation in dB between the peak and the neighboring cell towards the radar,
*f*_2_ is the separation in dB between the peak and the neighboring cell away from the radar.

The above constraints can be rewritten as:

$$3-f_{1}\leq 0$$

$$3-f_{2}\leq 0$$

which will generally not be convex. As the bandwidth changes, the size and position of the range gates change and the values of *f*_1_ and *f*_2_ change in an arbitrary fashion. However, if the gate containing the peak target is forced to be centered in the range dimension, then the above constraints can be approximated by convex functions. The accuracy with which the functions can represent the above constraints will greatly influence the correctness of the results.

The Lagrangian duality method was used to solve the problem [22]. The Lagrangian for our problem is:

(1) $$L(x,\lambda )=x+\sum_{i=1}^{2}\lambda_{i}(3-f_{i})$$

where *λ_i_* is the Lagrange multiplier associated with the *i*th inequality constraint. The Lagrange dual function *g* is the minimum value of the Lagrangian over *x*. If *D* represents the domain of the problem, then the dual function is expressed as [22]:

(2) $$g(\lambda )=\underset{x\in D}{\inf}L(x,\lambda )$$

The maximum of the dual function gives a lower bound on the optimum value of *x*.

### 3.2. Examples

A Target Scenario 1 is shown in Table 3. There are two targets separated by 2.5 m. A range resolution value of 2.5 m or better will be able to resolve the two targets, but this is only assuming that the point spread function is an ideal impulse. In reality, the point spread function is usually a Gaussian or a sinc-function having energy beyond its 3-dB point, which may cause a larger target to obscure a closer but smaller target. Thus, to be on the safe side, a resolution value of half of the minimum target separation is used to ensure capturing targets of all sizes and make target identification possible. Thus, in this case, a resolution of about 1.25 m would be ideal. Simulations were performed to find sample values for the constraints. From three values obtained by simulations, convex quadratic functions were approximated, as shown below, respectively:

(3) $$1.8\times 10^{-5}x^{2}-0.017x+1.482\leq 0$$

(4) $$5.3\times 10^{-6}x^{2}-0.0048x-3.5\leq 0$$

**Figure 4.**
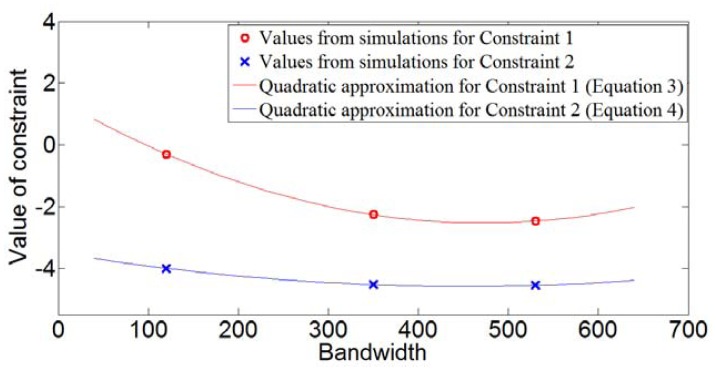
Representation of constraints by quadratic equations.

These constraints are plotted in Figure 4.

**Figure 5.**
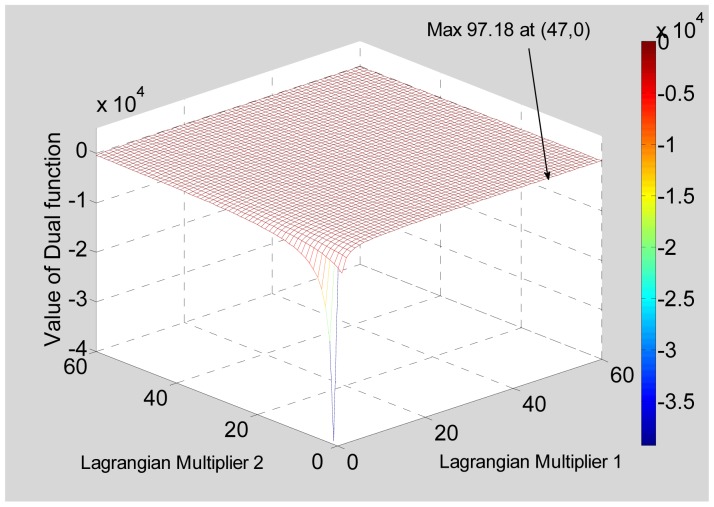
Dual function for a Target Scenario 1.

The dual function was obtained and its values for different values of Lagrangian multipliers are shown plotted in Figure 5. The maximum of the dual function has a value of 97.18 MHz, which corresponds to a range resolution of 1.54 m. This is close to the desired 1.25-m resolution, indicating that our technique works well.

**Figure 6.**
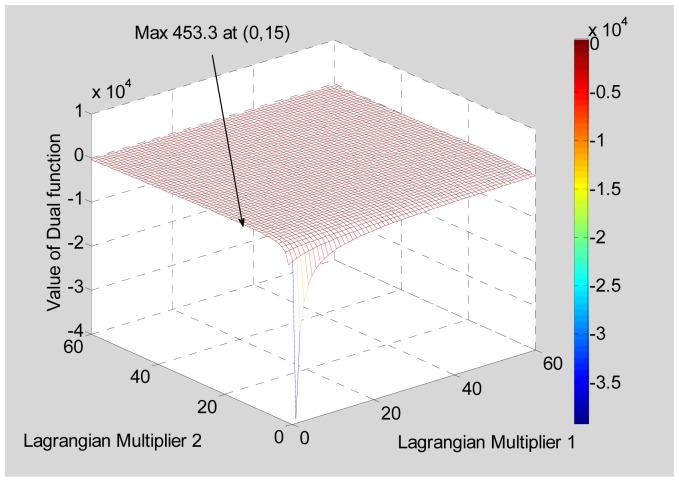
Dual function for a Target Scenario 2.

Another Target Scenario 2 is shown in Table 4. The minimum separation is 0.6 m, calling for a resolution of about 0.3 m. The maximum of the dual function has a value of 453.3 MHz corresponding to a range resolution of 0.33 m, which is again close to the desired value. The dual function is shown in Figure 6.

**Figure 7.**
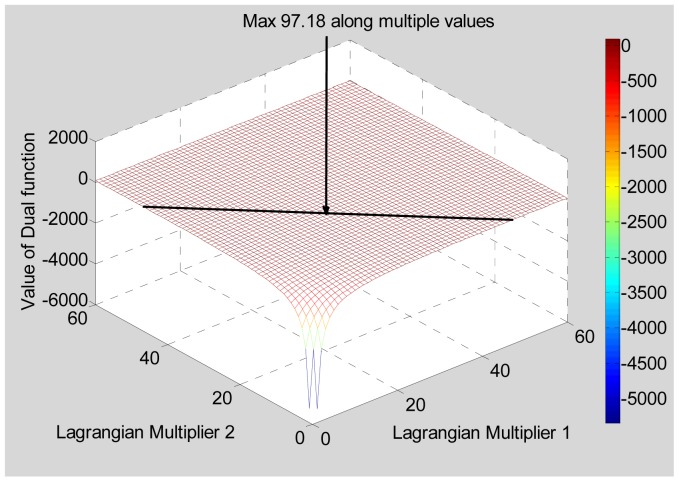
Dual function for a Target Scenario 3.

In the above two examples, we see that the maximum occurs at a value where either *λ*_1_ or *λ*_2_ is zero. This signifies that one of the constraints is dominant and is masking the other constraint. Hence a symmetrical Target Scenario 3 was tried, as shown in Table 5. This is similar to target scenario 1 except that another target is added to make the scene symmetrical. The optimum bandwidth is 97.18 MHz, which is the same as was obtained for Target Scenario 1. Here the maximum occurs at multiple values of pairs of (*λ*_1_, *λ*_2_), some of which have both *λ*_1_ and *λ*_2_ non-zero. The dual function for this target scenario is shown in Figure 7.

### 3.3. Bandwidth Requirement for Targets of Unequal Strength

**Figure 8.**
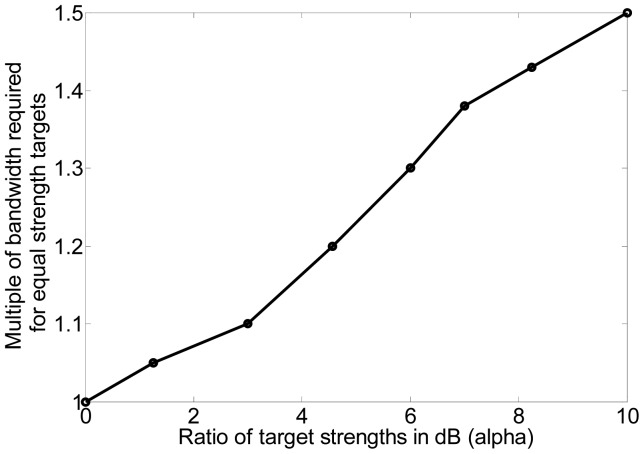
Dependance of bandwidth required for resolution on target strength ratio.

For the previous discussion, we assumed that the targets were of equal strength. However, if the targets vary greatly in strength, a resolution value of half of the target separation is no longer accurate. In [20], the effect of both bandwidth and the difference in target RCS on range resolution was examined. We carried out simulations to find a relationship between the strength difference *α* (in dB) and the bandwidth requirement. This is plotted in Figure 8 and it can be seen that the relationship is almost linear. The ratio of target strengths is expressed in dB. The change in bandwidth is expressed as the multiple of bandwidth required for equal strength targets. Since the bandwidth increases by 50% (0.5) for a 10-dB increase in *α*, the slope of the straight line approximation is 0.5/10 = 1/20. Hence, for targets of unequal strength, the bandwidth optimization problem can be stated as:

minimize *x*
subject to *f*_1_(*x*, *α*) ≥ 3 dB where *x* is the bandwidth used for a target scene,
  *α* is the ratio of target strengths in dB (*α* ≥ 0),
  *f*_1_ is the separation in dB between the cell containing the weaker target and the neighboring cell towards the stronger target.

To solve the above problem, we first solve the simpler problem using *α* = 0 and arrive at the solution *β* using the procedure introduced before. The actual bandwidth *β_req_* for the problem can then be arrived at using the expression in Equation (5), wherein the terms 0.5 and 10 come from the slope of the straight line approximation described earlier:

(5) $$\beta_{\text{req}}=\beta (1+\frac{0.5\alpha }{10})=\beta (1+\frac{\alpha }{20})$$

**Figure 9.**
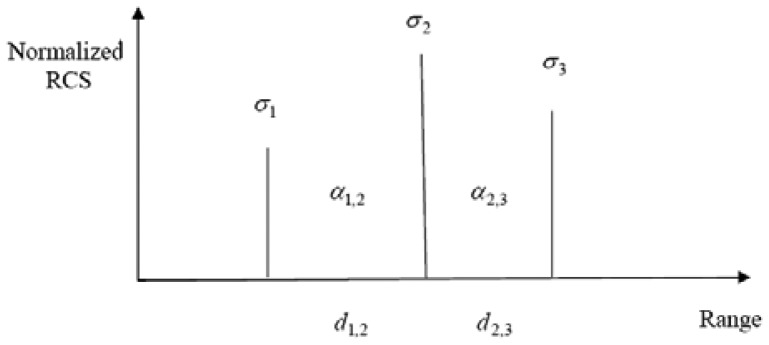
Scattering centers of an extended target.

Let us consider an arbitrary target shown in Figure 9 and calculate the bandwidth required for its detection. For each pair of adjacent scattering centers, we take the ratio of the RCS of the stronger one to the weaker one to get *α* in dB. We separately look at the bandwidth required for resolving each pair of adjacent scattering centers. Let us first look at the pair (*σ*_1_, *σ*_2_). We approximate the expected value of bandwidth based on the theory of point spread function. If *σ*_1_ and *σ*_2_ were equal, the resolution required would have been approximately *d*_1,2_/2. This corresponds to a bandwidth of *β*_1.2_ = *c*/*d*_1.2_. When *σ*_1_ and *σ*_2_ are unequal, this can be adjusted to be equal to:

(6) $$\beta_{1,2\text{req}}=\frac{c}{d_{1,2}}\left(1+\frac{\alpha_{1,2}}{20}\right)$$

Looking at the other pairs of scattering centers, for a total of *n* scattering centers, the bandwidth required can be expressed as:

(7) $$\beta_{\text{req}}=\underset{i\in (1,n-1)}{\max}\left[\frac{c}{d_{i,i+1}}\left(1+\frac{\alpha_{i,i+1}}{20}\right)\right]$$

When we consider just the pair of adjacent scattering centers, we neglect the effect of other scatterers. This can be justified from the fact that if a scatterer were close enough to make a substantial contribution, then the bandwidth required to resolve that scatterer from its neighbor would be much higher than the others in the expression above.

## 4. Field Measurement Results

**Figure 10.**
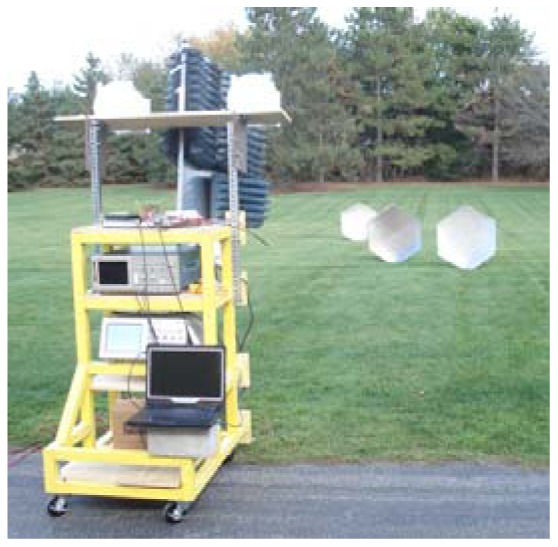
Field measurement setup for multimodal radar.

Field measurements were performed for a number of different scenarios. The measurement setup is shown in Figure 10. Trihedral corner reflectors with square faces of length 0.6096 m (2 ft) were used as targets whose computed RCS ranged from 57.8 m^2^ (+17.6 dBsm) at 1 GHz to 155.5 m^2^ (+21.9 dBsm) at 1.64 GHz. We consider a range of 37.5 m which is slightly greater than the maximum radar range. This results in 10 LRR gates each of extent of 3.75 m. The RCS values of the targets are normalized with respect to the target with the highest RCS. The algorithm continues with higher resolution passes until 3-dB separations are obtained between the peaks and their neighboring cells.

### 4.1. Staring Mode

**Figure 11.**
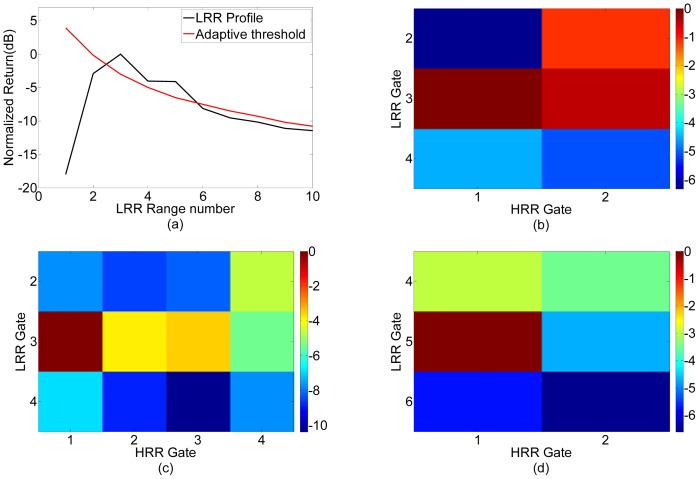
Imaging results for Target Scenario 4. (**a**) Pass 1. (**b**) Pass 2, LRR Gate 3. (**c**) Pass 3, LRR Gate 3. (**d**) Pass 2, LRR Gate 5.

The following results were obtained when the multimodal radar system was operated in the staring mode. Table 6 shows a Target Scenario 4 with three targets. A subset of this scenario was seen earlier in Target Scenario 1 considered for bandwidth optimization. The LRR pass correctly identifies LRR Gates 3 and 5 for further processing. The multimodal algorithm runs and stops at Pass 3 for LRR Gate 3 and Pass 2 for LRR Gate 5, as shown in Figure 11. LRR Gate 3 contains two targets separated by 2.5 m. A resolution of around 1.25 m would be ideal. The multimodal radar required a bandwidth of 160 MHz for this target separation which corresponds to a resolution of 0.93 m. This is close to the expected result. Since a bandwidth of 80 MHz is not sufficient to resolve these targets, we do not see sufficient separation between cells in Pass 2 in Figure 11(b). Pass 3 gives us 3-dB separation as seen in Figure 11(c) and the algorithm stops. The optimization process in Section III yielded a bandwidth of 97.14 MHz. The higher bandwidth in the field can be attributed to the large size of the corner reflectors and other effects such as ground reflection. LRR Gate 5 contained a single target which resulted in the multimodal radar stopping at the first higher resolution pass of 80 MHz as shown in Figure 11(d).

**Figure 12.**
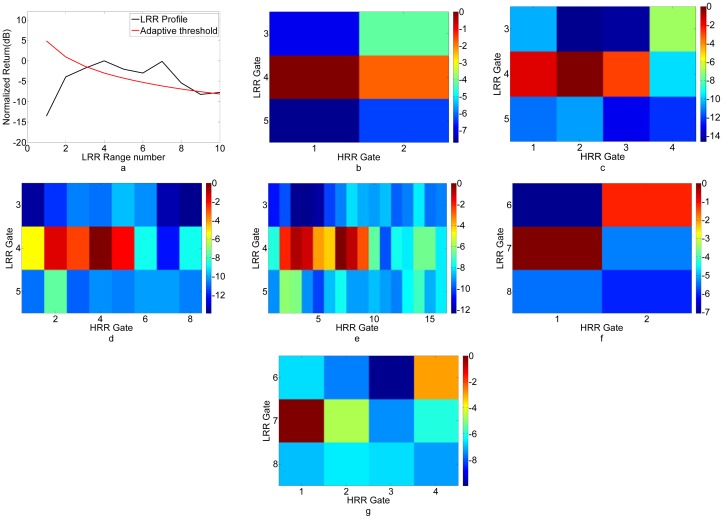
Imaging results for Target Scenario 5. (**a**) Pass 1. (**b**) Pass 2, LRR Gate 4. (**c**) Pass 3, LRR Gate 4. (**d**) Pass 4, LRR Gate 4. (**e**) Pass 5, LRR Gate 4. (**f**) Pass 2, LRR Gate 7. (**g**) Pass 3, LRR Gate 7.

Table 7 shows a Target Scenario 5 with three targets. A subset of this scenario was seen earlier in Target Scenario 2 considered for bandwidth optimization. A relative RCS of 4 (target scene number 2) indicates that four corner reflectors were placed side-by-side in that particular range cell. Here the algorithm stops at Pass 5 for LRR Gate 4 and Pass 3 for LRR Gate 7, as shown in Figure 12. The targets in LRR Gate 4 are separated by 0.6 m. A resolution of around 0.3 m is required for differentiating these targets. The multimodal algorithm uses the entire bandwidth of 640 MHz corresponding to 0.23-m resolution for this target scene. This is also close to the expected result. As seen in Figure 12(b) to Figure 12(d), none of the HRR passes from 2 to 4 is able to resolve these targets. HRR pass 5 clearly discerns the two targets in Figure 12(e). In Section III, the optimization process yielded a bandwidth of 453.3 MHz for the same scenario. The bandwidth in the field is again more than the theoretically optimized bandwidth because of the reasons mentioned before. For LRR Gate 7, we had multiple corner reflectors placed at the same range to act as a single target. The radar required a bandwidth of 160 MHz for resolving this target as shown in Figure 12(g). While this is more than the expected bandwidth of 80 MHz for a single target, it may be due to the fact that our corner reflectors are not point targets and may happen to be split across two gates.

### 4.2. Scanning Mode

**Figure 13.**
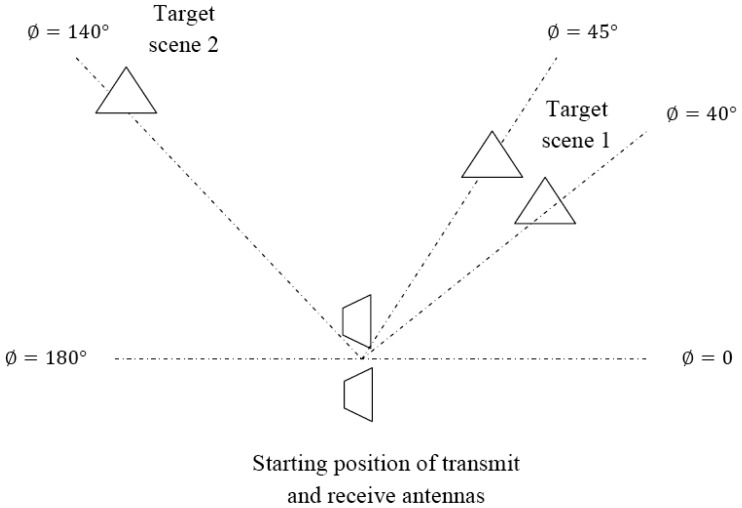
Diagrammatic representation of Target Scenario 6.

**Figure 14.**
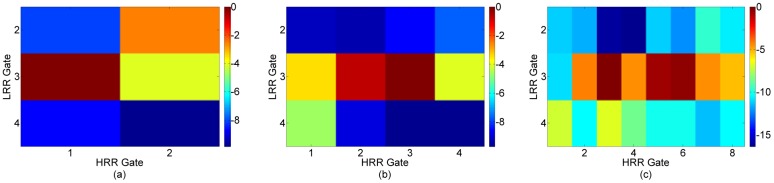

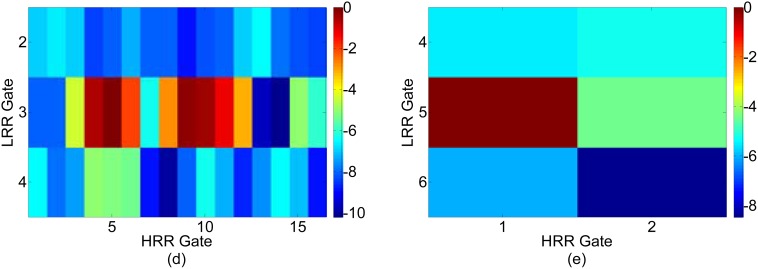
HRR images for Target Scenario 6. (**a**) Pass 2, Target scene 1. (**b**) Pass 3, Target scene 1. (**c**) Pass 4, Target scene 1. (**d**) Pass 5, Target scene 1. (**e**) Pass 2, Target scene 2.

Table 8 lists a Target Scenario 6 consisting of two target scenes. It is shown diagrammatically in Figure 13. LRR profiles are taken while the antennas are rotated with a step-size of 15°. The targets are correctly picked out from the LRR tracks and the multimodal algorithm is applied to these. The HRR images for these target scenes are shown in Figure 14. For target scene 1, LRR Gate 3 contains two targets separated by 1 m. HRR passes 2 and 3 are not able to resolve these targets, as shown in Figure 14(a,b). However, the two targets are discerned in HRR passes 4 and 5 in Figure 14(c,d). For target scene 2, HRR pass 2 is able to resolve the single target in LRR gate 5, as shown in Figure 14(e). This illustrates how the multimodal radar can be used to scan an area for targets and use only as much bandwidth as absolutely required.

**Figure 15.**
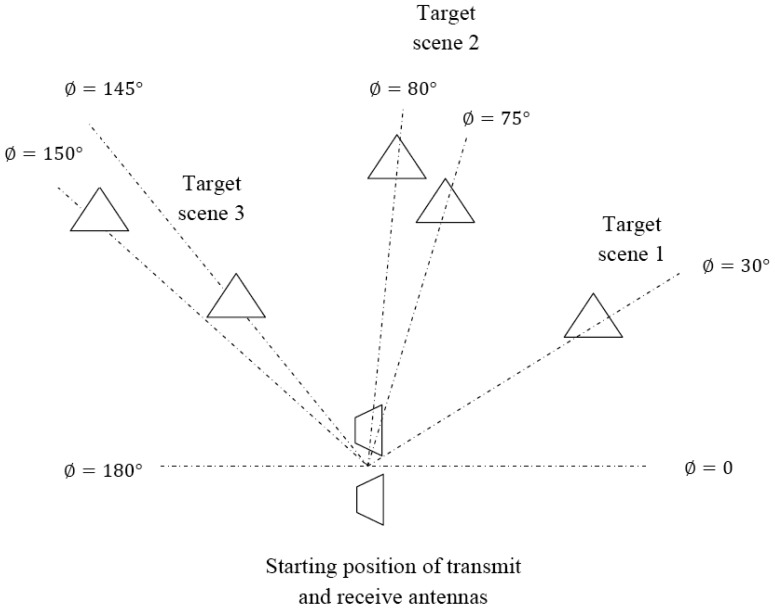
Diagrammatic representation of Target Scenario 7.

**Figure 16.**
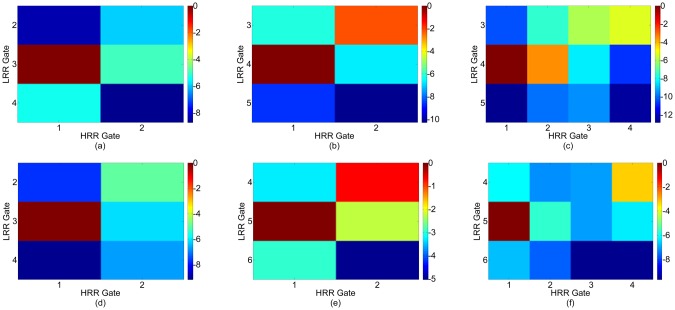
HRR Images for a Target Scenario 7. (**a**) Pass 2, Target scene 1. (**b**) Pass 2, Target scene 2. (**c**) Pass 3, Target scene 2. (**d**) Pass 2, Target scene 3, LRR gate 3. (**e**) Pass 2, Target scene 3, LRR gate 5. (**f**) Pass 3, Target scene 3, LRR gate 5.

Table 9 describes a Target Scenario 7 consisting of three target scenes which are shown in Figure 15. The HRR images for this target scenario are shown in Figure 16. Target scene 1 consists of a single target and is resolved by pass 2 as shown in Figure 16(a). Target scene 2 consists of two targets separated by 0.9 m. These 2 targets are discerned in Pass 3 as shown in Figure 16(c). Target scene 3 has one target each in LRR Gate 3 and 5. The target in LRR Gate 3 is resolved in Pass 2 as shown in Figure 16(d). The target in LRR Gate 5 in resolved in Pass 3 as shown in Figure 16(f).

### 4.3. Targets of Unequal RCS

**Figure 17.**
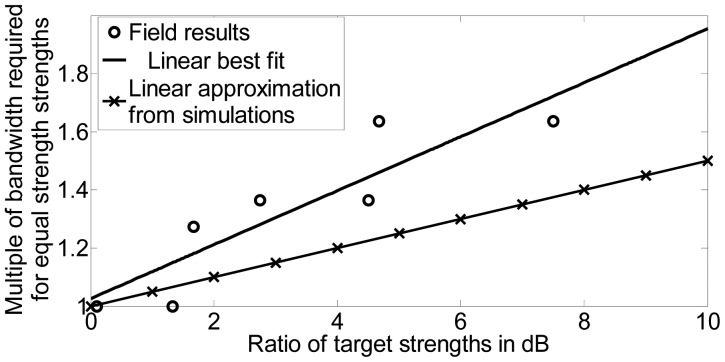
Dependance of bandwidth on target strength ratio.

Field data were acquired to study the variation in bandwidth required based on difference in target strength. Multiple corner reflectors were placed side by side to increase the resultant RCS. Table 10 shows the various target scenes and the bandwidth required for each scene. Note that the RCS of second target is normalized with respect to its strength and distance compared to the first target. The results are plotted as shown in Figure 17. The linear approximation derived from simulation results, *i.e.*, the line corresponding to Equation (5) is shown. The results from the field are shown and a best fit line is drawn. Clearly, the field results demonstrate that higher bandwidth is required to resolve two targets as the ratio of their strengths increases. Hence our theoretical results in this regard are justified. We did require slightly more bandwidth in the field compared to the simulation results. This may be due to the fact that we are using multiple corner reflectors to act as a single target leading to a broader response and hence requiring more bandwidth for 3-dB separation. Effects such as ground reflection also result in a wider response than a point target. Also, since multiple corner reflectors are placed side by side, the gain of the antenna would vary for the different reflectors. Furthermore, some of the response of the farther reflectors is masked by the reflectors in front.

**Figure 18.**
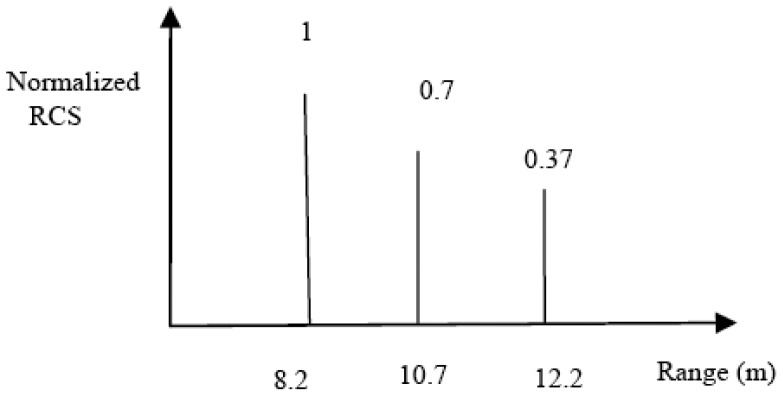
An extended target using corner reflectors.

An extended target was simulated by placing corner reflectors at appropriate positions. One corner reflector is placed at 8.2 m, and two each at 10.7 m and 12.2 m. After normalizing with respect to strength and distance, the resulting extended target is as shown in Figure 18. Using Equation (7), the required bandwidth comes out to be 136.6 MHz. In the field, for the above scenario, the required bandwidth came out to be 190 MHz. This is higher than the theoretically expected bandwidth due the same reasons stated earlier.

## 5. Observations from Simulations

In this section, we look at certain results obtained from simulations. We also try to understand how the number of passes required by the multimodal radar would change with respect to external conditions.

### 5.1. Receiver Operating Characteristics

Simulations were performed to generate the receiver operating characteristics (ROC) curves for the multimodal radar. The probability of detection (*P_d_*) and probability of false alarm (*P_f_*) for a multimodal radar can be defined as shown below:

(8) $$P_{d}=\frac{\text{No of targets correctly forecast in every pass}}{\text{Total number of targets}}$$

(9) $$P_{f}=\frac{\text{No of targets that were falsely forecast in all passes}}{\text{Total number of non}-\text{target cells}}$$

Different targets may experience different number of passes. The *P_f_* varies depending on the number of passes executed. It is proposed that the *P_f_* for the multimodal radar be defined as the average of the values obtained for the different passes:

(10) $$P_{f}=\frac{P_{f(2)}+P_{f(3)}+P_{f(4)}+P_{f(5)}}{4}$$

**Figure 19.**
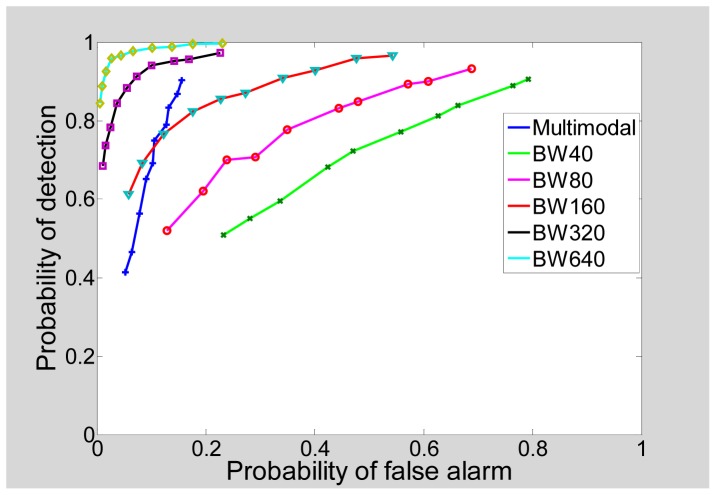
Receiver operating characteristics.

Figure 19 shows the ROC of the multimodal radar and compares it with the ROC plots of single pass radars of various bandwidths. As expected, for a given probability of false alarm (*P_f_*), the probability of detection (*P_d_*) improves as bandwidth increases. We also note that the *P_f_* of the multimodal radar does not deteriorate too rapidly with the increase in the *P_d_*, as is seen for the single pass radars. This is expected since the existence of multiple passes reduced the chances of false alarm as compared to single pass radars. Also, the *P_d_* of the multimodal radar is limited by the *P_d_* of its LRR pass. This follows from the fact that the LRR pass is the first pass in the multimodal radar and any target missed in this pass goes undetected in the higher bandwidth passes which focus only on the LRR gates found earlier. Hence, the *P_d_* of the multimodal radar may be improved by selecting an initial LRR pass with higher bandwidth.

### 5.2. Effect of Separation between Targets

**Figure 20.**
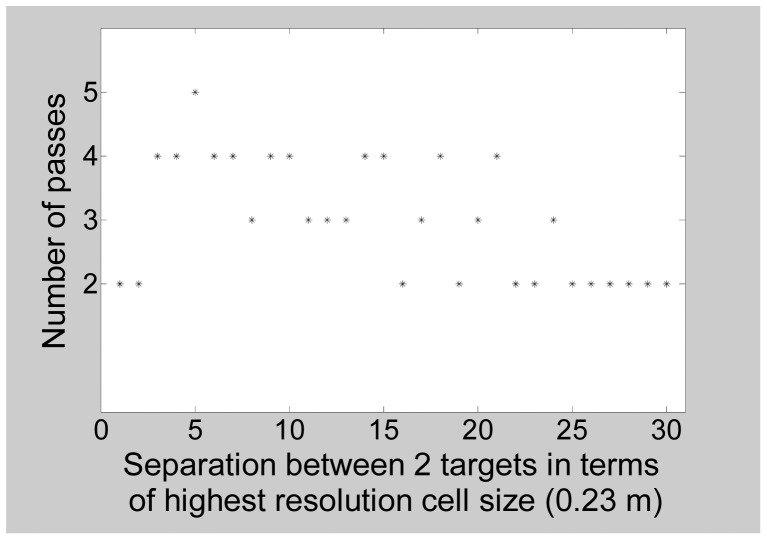
Variation in required number of passes as a function of target separation.

We explore the effect of separation between the targets. We consider two targets and note the number of passes required as the separation between them is gradually reduced. The result is plotted in Figure 20 where the X-axis is in terms of the highest resolution cell size (0.23 m). The number of passes required generally increases as the separation between the targets is reduced. Hence more bandwidth would be utilized as the distance between two targets is reduced. This suggests that as the target scene becomes more intricate, the multimodal radar would require more number of passes. The number of significant peaks observed in Pass 2 HRR images can also provide an indication of an intricate target scene. An important consideration is whether an attempt should be made to skip some intermediate bandwidths and directly advance to a higher bandwidth for such target scenes. Figure 20 shows that when the target separation was too low, the multimodal radar took only two passes. This happened because in Pass 2, the two targets appeared as a single target.

### 5.3. Effect of Signal-to-Noise Ratio

**Figure 21.**
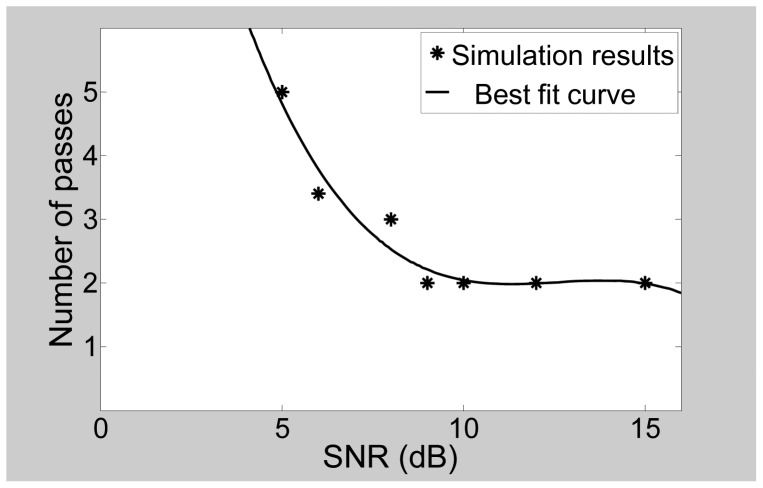
Variation in required number of passes as a function of SNR.

We also simulated the effect of signal-to-noise ratio (SNR) on the performance of the multimodal radar. The target scenario was kept the same while the SNR was varied. It was observed that a higher number of passes is required to resolve a target scene as the SNR is decreased. The result is plotted in Figure 21. This follows from the fact that better separations are obtained between the peaks and their neighboring cells at higher SNRs. As the SNR reduces, more passes are required to get adequate separation. At high SNRs, the number of passes levels off to a value of two for the scenarios under consideration, and starts to dip at an SNR of 15 dB. It is possible that for certain scenarios at high SNR values, a single pass may be adequate to resolve the target scene.

## 6. Conclusions

The methodology of a multimodal radar system with progressive resolution enhancement is described. This radar makes it possible to look at different target scenes with the appropriate bandwidth required to resolve the target features. The saved bandwidth can be made available for use by other applications. Experimental results were provided to give a demonstration of the multimodal radar algorithm in operation. Simulation results were shown to provide further insight into the performance characteristics of the radar. A theoretical method was discussed to optimize the bandwidth required by the multimodal radar. It was seen that this bandwidth increases significantly when the targets differ greatly in strength.

Several other considerations also come to mind. For example, there may be an overriding need to reserve a significant portion of the available spectrum for other applications, such as essential communications. In such a case, there may be an upper limit to the bandwidth available for the radar. In addition, if a specific smaller portion of the spectrum needs to be reserved for alternate applications, the radar may need to search for available contiguous spectrum for its operation within the entire band while avoiding the reserved subband. These issues require additional study. With the advent of software-defined RF technology, future multimodal radar systems can be designed to be reconfigurable, and therefore highly flexible and adaptive [23].

## Figures and Tables

**Table 1.** Radar system parameters.

**Parameter**	**Value**
Radar waveform	Linear frequency modulated pulse
Radar bandwidth	40–640 MHz
Pulse width	16 *μ*s
Transmit power	Approx. 0.5 W
Maximum radar range	Approx. 25 m

**Table 2.** Bandwidth and resolution for each pass of the multimodal radar.

**Pass**	**Bandwidth (MHz)**	**Resolution (m)**
1	40	3.75
2	80	1.87
3	160	0.93
4	320	0.46
5	640	0.23

**Table 3.** Target Scenario 1.

**Target Number**	**Range (m)**	**Relative RCS**
1	8.2	1
2	10.7	1

**Table 4.** Target Scenario 2.

**Target Number**	**Range (m)**	**Relative RCS (sq. m)**
1	11.5	1
2	12.1	1

**Table 5.** Target Scenario 3.

**Target Number**	**Range (m)**	**Relative RCS**
1	8.2	1
2	10.7	1
3	13.2	1

**Table 6.** Target Scenario 4.

**Target Number**	**Range (m)**	**LRR Gate**	**Relative RCS**
1	8.2	3	1
2	10.7	3	1
3	15.8	5	1

**Table 7.** Target Scenario 5.

**Target Number**	**Range (m)**	**LRR Gate**	**Relative RCS**
1	11.5	4	1
2	12.1	4	1
3	22.1	7	4

**Table 8.** Target Scenario 6.

**Target Scene Number**	***ϕ* (Degrees)**	**Range (m)**	**LRR Gate**	**Relative RCS**
1	40	8.5	3	2
	45	9.5	3	2
2	140	15.8	5	4

**Table 9.** Target Scenario 7.

**Target Scene Number**	***ϕ* (degrees)**	**Range (m)**	**LRR Gate**	**Relative RCS**
1	30	8.2	3	1
2	75	11.3	4	1
	80	12.2	4	1
3	145	8.2	3	1
	150	15.2	5	2

**Table 10.** Field results for Scenario WITH varying relative RCS

**No. of Corner Reflectors at 8.2 m**	**No. of Corner Reflectors at 10.7 m**	**Normalized RCS of Second Target Compared to First Target**	**Ratio of Stronger Target To Weaker Target in dB**	**Bandwidth Required for 3 dB Separation for Each Peak**
1	1	0.345	4.62	180
1	2	0.69	1.62	140
1	3	1.035	0.15	110
1	4	1.38	1.4	110
2	1	0.172	7.62	180
2	2	0.345	4.62	150
2	3	0.518	2.86	150
